# Supplementary material for: Anticancer Activity of (S)-5-Chloro-3-((3,5-dimethylphenyl)sulfonyl)-N-(1-oxo-1-((pyridin-4-ylmethyl)amino)propan-2-yl)-1H-indole-2-carboxamide (RS4690), a New Dishevelled 1 Inhibitor
Source: Cancers (Basel). 2022 Mar 7;14(5):1358. doi: 10.3390/cancers14051358 (PMC8909805; doi:10.3390/cancers14051358)
Supplement: Supplementary file 1 [file cancers-14-01358-s001.zip › cancers-1611255-supplementary.pdf]

Supplementary Materials

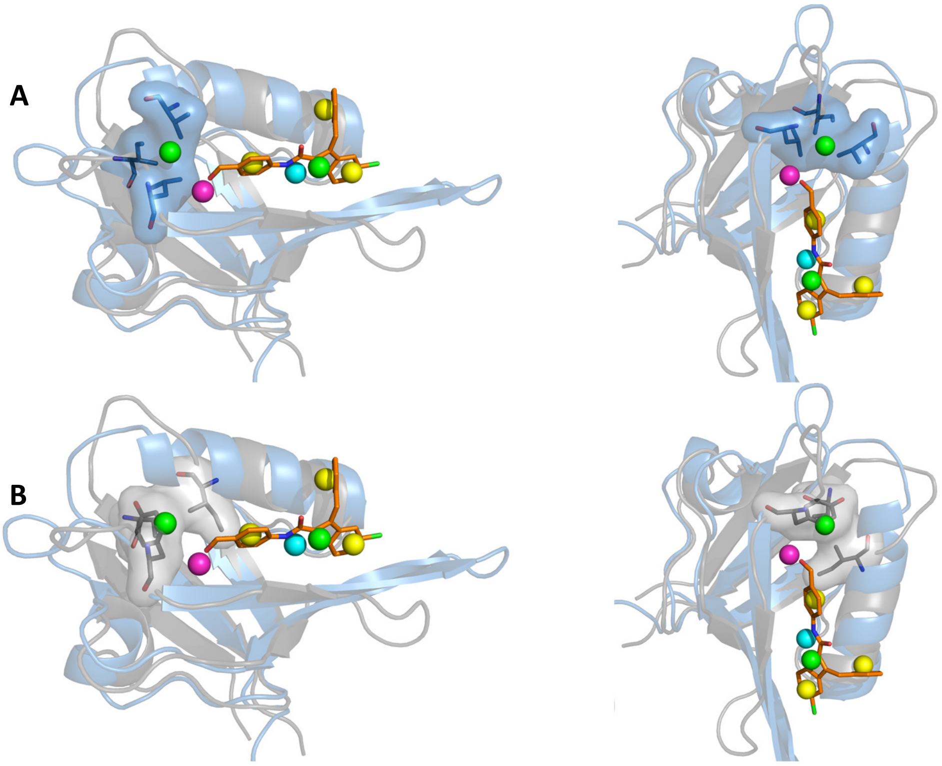

Figure S1. Map of the pharmacophore features superimposed with the **1** binding mode. The model has 7 features: 3 aromatic (yellow), 1 H-bond acceptor (magenta), 1 H-bond donor (cyan;) and 2 hydrophobic (green). The PDZ domains are also reported: light blue for DVL1 PDZ and grey for NHERF1. The residues Leu12, Val75 and Ile81 that sized an additional sub-pocket for DVL1 are depicted as light blue stick and surface (panel **A**). The counterpart for NHERF1 DVL1 (panel **B**) is reported in grey stick and surface. The picture reported a frontal view (left side) and to better appreciate the space sized by the smaller residues of DVL1 PDZ a rotated (90 degrees) view (right side).

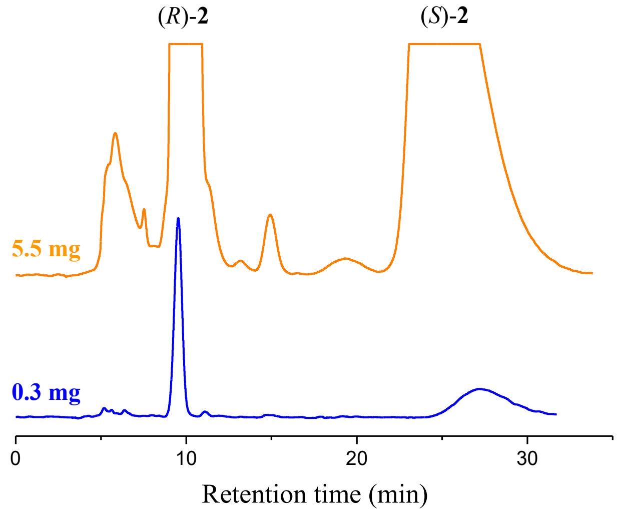

Figure S2. Loading study of racemate **2** on the Chiralpak AD CSP on a semipreparative scale. Chromatographic conditions: mobile phase, n-hexane–ethanol-DEA 10:90:0.1 (*v*/*v*/*v*); flow-rate, 3.0 ml/min; temperature, 40 °C; detection, UV at 254 nm; injection volume, 0.1 mL (orange line) and 2.0 mL/min (blue line).

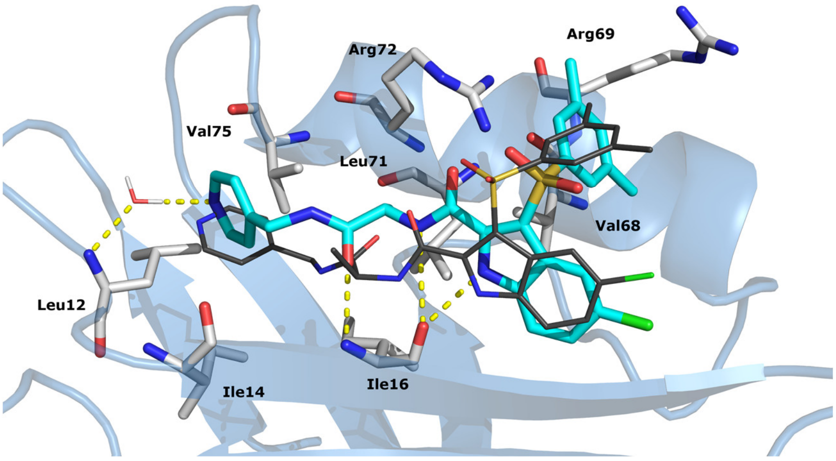

Figure S3. Snapshot of the trajectory of compound (*S*)-**1** with DVL1 PDZ. The docking pose of (*S*)-**1** s reported as grey lines; residues involved in interactions were reported as white stick; H-bonds are depicted as yellow dot lines.Analyses of the trajectory showed two additional H-bonds, both with the Ile16 backbone: one involving the nitrogen atom of the first carboxamide group and another involving the oxygen atom of the second amide moiety Furthermore, the sulfone moiety was at bond distance from Arg72. The Arg69 residue moved away from the dimethyl phenyl moiety doing polar contacts with Glu65 (not shown in the picture). The chiral methyl group pointed toward β2 helix, forming hydrophobic contacts in a small cleft that accommodated the side chain of the residues -2 of the cognate substrate. It was also observed a water molecule that bridged the pyridine nitrogen atom with the Leu12 backbone.

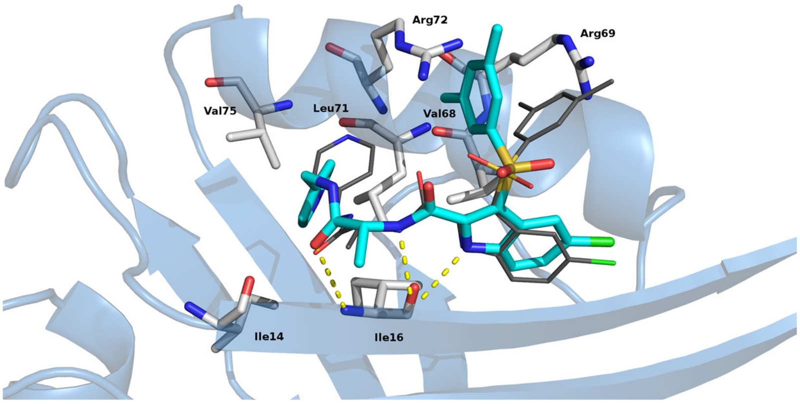

Figure S4. Snapshot of the trajectory of compound (*R*)-**1** with DVL1 PDZ. The docking pose of (*R*)-**1** is reported as grey lines; residues involved in interactions were reported as white stick; H-bonds are depicted as yellow dot lines. The analyses of the trajectory showed that the interactions highlighted by docking experiments were stable during the simulation time Also, for the R-enantiomer it was observed a new H-bond between the carboxylic oxygen atom and the Ile16 backbone. The chiral methyl group pointed toward the solvent exposed area reducing the quality of the binding.
